# The p.Arg435His Variation of IgG3 With High Affinity to FcRn Is Associated With Susceptibility for Pemphigus Vulgaris—Analysis of Four Different Ethnic Cohorts

**DOI:** 10.3389/fimmu.2018.01788

**Published:** 2018-08-02

**Authors:** Andreas Recke, Sarah Konitzer, Susanne Lemcke, Miriam Freitag, Nele Maxi Sommer, Mohammad Abdelhady, Mahsa M. Amoli, Sandrine Benoit, Farha El-Chennawy, Mohammad Eldarouti, Rüdiger Eming, Regine Gläser, Claudia Günther, Eva Hadaschik, Bernhard Homey, Wolfgang Lieb, Wiebke K. Peitsch, Claudia Pföhler, Reza M. Robati, Marjan Saeedi, Miklós Sárdy, Michael Sticherling, Soner Uzun, Margitta Worm, Detlef Zillikens, Saleh Ibrahim, Gestur Vidarsson, Enno Schmidt, the German AIBD Genetic Study Group, Alexander Kreuter

**Affiliations:** ^1^Lübeck Institute of Experimental Dermatology (LIED), University of Lübeck, Lübeck, Germany; ^2^Department of Dermatology, Allergology and Venereology, University of Lübeck, Lübeck, Germany; ^3^Department of Dermatology, Faculty of Medicine, Cairo University, Giza, Egypt; ^4^Metabolic Disorders Research Center, Endocrinology and Metabolism Molecular – Cellular Sciences Institute, Tehran University of Medical Sciences, Tehran, Iran; ^5^Department of Dermatology, Venereology and Allergology, University Hospital Würzburg, Würzburg, Germany; ^6^Department of Clinical Pathology, Mansoura Faculty of Medicine, Mansoura University, Mansoura, Egypt; ^7^Department of Dermatology and Allergology, Phillips-Universität Marburg, Marburg, Germany; ^8^Department of Dermatology, Venereology and Allergology, Christian Albrecht University, Kiel, Germany; ^9^Department of Dermatology, University Hospital of Dresden, Dresden, Germany; ^10^Department of Dermatology, Ruprecht-Karls-University of Heidelberg, Heidelberg, Germany; ^11^Department of Dermatology, Heinrich Heine University, Düsseldorf, Germany; ^12^Institute of Epidemiology, Christian-Albrechts-University, Kiel, Germany; ^13^Popgen Biobank, Christian-Albrechts-University, Kiel, Germany; ^14^Department of Dermatology, University Medical Center Mannheim, Heidelberg University, Mannheim, Germany; ^15^Department of Dermatology, Vivantes Klinikum im Friedrichshain, Berlin, Germany; ^16^Department of Dermatology, Saarland University Medical School, Homburg/Saar, Germany; ^17^Skin Research Center, Shahid Beheshti University of Medical Sciences, Tehran, Iran; ^18^Department of Dermatology, Ludwig Maximilian University Munich, Munich, Germany; ^19^Department of Dermatology, University of Erlangen-Nuremberg, Erlangen, Germany; ^20^Department of Dermatology, Faculty of Medicine, Akdeniz University, Antalya, Turkey; ^21^Department of Dermatology, Venerology and Allergology, Allergy Center Charité, Charité-Medical University Berlin, Berlin, Germany; ^22^Department of Experimental Hematology, Sanquin Research Institute, Amsterdam, Netherlands

**Keywords:** immunology, dermatology, autoantibodies, allotype, pemphigus, pemphigoid, half-life, functional genetics

## Abstract

IgG3 is the IgG subclass with the strongest effector functions among all four IgG subclasses and the highest degree of allelic variability among all constant immunoglobulin genes. Due to its genetic position, IgG3 is often the first isotype an antibody switches to before IgG1 or IgG4. Compared with the other IgG subclasses, it has a reduced half-life which is probably connected to a decreased affinity to the neonatal Fc receptor (FcRn). However, a few allelic variants harbor an amino acid replacement of His435 to Arg that reverts the half-life of the resulting IgG3 to the same level as the other IgG subclasses. Because of its functional impact, we hypothesized that the p.Arg435His variation could be associated with susceptibility to autoantibody-mediated diseases like pemphigus vulgaris (PV) and bullous pemphigoid (BP). Using a set of samples from German, Turkish, Egyptian, and Iranian patients and controls, we were able to demonstrate a genetic association of the p.Arg435His variation with PV risk, but not with BP risk. Our results suggest a hitherto unknown role for the function of IgG3 in the pathogenesis of PV.

## Introduction

**Figure 1:**
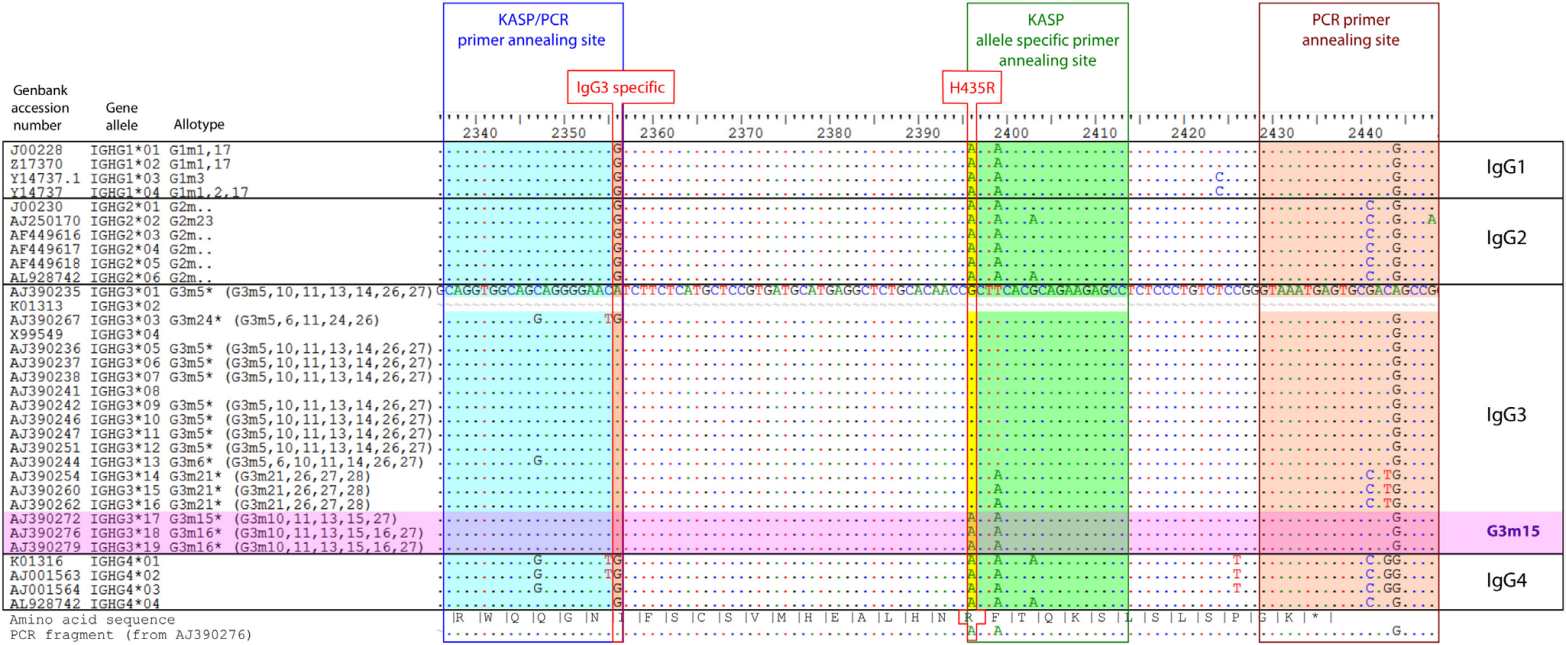
Alignment of human IgG gene alleles. Alignment of all human IgG alleles listed in IMGT [http://www.imgt.org (Accessed: 15 March, 2015)], including GenBank accession number, gene names, isotype, allele numbering, and allotype numbering. Primer annealing sites for KASP and IgG3-specific PCR are shaded in blue, green, and red, respectively. Nucleotides unique in IgG3 (IgG3 specific) and the g.1053927G>A variation (p.Arg435His) are indicated with a red border. The full DNA sequence is shown for the reference sequence (AJ390235), for all other sequences, all bases except those that differ from the reference are masked by a dot. The amino acid sequence of the reference sequence is shown on the bottom site, together with the PCR fragment amplified for Sanger sequencing. The IGHG3 alleles that contain the g.1053927G>A variation are highlighted in pink (G3m15). A marker for DNA positions within the alignment set is given on the top scale.

Of the four human IgG subclasses, IgG3 (gene IGHG3) exhibits the strongest capabilities to activate complement and effector cells of the innate immune system (1). Compared with the other IgG subclasses, IgG3 has a reduced half-life which is probably caused by a decreased affinity to FcRn (2). FcRn is involved in IgG transport across mucosal cells and the placenta. Moreover, FcRn is responsible for the exceptionally long half-life of IgG, and it rescues IgG from lysosomal degradation by recycling it back to the surface in endothelial cells. It further appears to be involved in IgG-mediated phagocytosis (3). The reduced half-life of IgG3 appears to be caused by an amino acid replacement at residue 435, where this IgG subclass typically carries an arginine instead of a histidine like the other IgG subclasses. From the 19 different alleles that the IMGT (ImMunoGeneTics information) database^1^ lists for IGHG3, 3 alleles contain a histidine instead of an arginine at this position (IgG3:p.Arg435His variation, NG_001019.5:g.1053927G>A, RefSNP rs4042056, Figure 1). These three alleles are associated with the G3m15 and G3m16 allotypes which are quite rare in the European population and more frequent in Asia and Africa (4). The histidine residue at position 435 leads to a higher affinity of IgG3 to FcRn and, consequently, to a prolonged half-life and most likely to an increased bioavailability in extravascular tissues (2).

Immunoglobulin allotypes represent serological features of genetic variations within the constant domains. Gm–Am allotypes are inherited as fixed combination, also termed Gm–Am haplotypes (5). They have been associated with various autoimmune diseases and different immune responses to bacterial infections (6). Of note, an association with immunoglobulin kappa light chain allotypes (Km allotypes) has been described for bullous pemphigoid (BP) but not for pemphigus vulgaris (PV) (7). However, the underlying mechanisms remain unexplained. Genetic polymorphisms with functional impact like the p.Arg435His variation of IgG3 might affect the etiology and pathogenesis of associated diseases, including autoantibody-mediated diseases. Therefore, this study was conducted to elucidate a possible association of the p.Arg435His variation with PV. PV is a severe autoimmune blistering disorder of the skin and surface-close mucous membranes, characterized by autoantibodies against desmosomal proteins of the epidermis and epithelia of mucosal membranes: desmoglein 3 and, in the mucocutaneous variant of PV, also desmoglein 1. IgG1 and IgG4, the most frequent IgG subclasses, have been demonstrated to cause acantholysis (8, 9). IgG3, however, is often the first IgG subclass that appears immediately after the B cell switch from IgM (10). IgG anti-Dsg3 autoantibodies lead to steric hindrance in desmoglein interaction, altered cellular signaling, and aberrant cellular distribution of desmogleins resulting in acantholysis, the formation of flaccid blisters and erosions (11, 12). The events that lead to the irregular production of autoantibodies are largely unknown. While PV is known to be strongly associated with certain HLA alleles, such as DRB1*0402 and DQB1*0503, we have recently described an association with the first non-HLA gene, ST18, in PV patients from Egypt and Israel, but not from Germany (13, 14). Moreover, PV susceptibility was found to be associated with certain polymorphisms of FcγRIIb and FcγRIIc, indicating a role of peripheral tolerance in this disease (15). Here, we followed up on the idea of non-HLA susceptibility genes in pemphigus and investigated a possible association with the functionally important p.Arg435His variation of IgG3.

## Materials and Methods

### Description of Cohorts

Samples and controls were stored and analyzed in the order in which they were collected, irrespective of age and gender. The inclusion criteria for PV and BP followed the recent guidelines of the German Society of Dermatology and European Academy of Dermatology and Venereology (16–18). In brief, PV was diagnosed based on the clinical picture with flaccid blisters and/or erosions on the skin and/or surface-close mucous membranes, intercellular IgG and/or C3 deposition in the epidermis/epithelia by direct immunofluorescence and/or detection of circulating anti-desmoglein 3 autoantibodies. In BP, diagnosis was made in patients with compatible clinical picture, i.e., tense blisters, erosions, prurigo-type lesions, and urticarial or eczematous lesions (19), deposition of IgG and/or C3 at the dermal–epidermal junction by direct immunofluorescence microscopy, and/or detection of serum BP180 NC16A IgG. The respective diagnosis was re-evaluated in the Department of Dermatology, Lübeck, Germany. Normal healthy controls were defined not to match any of the above criteria.

### Allotype-Specific ELISA

The G3m15 allotype was detected with a sandwich ELISA using the same single chain Fv (scFv) for capture and detection, as described (2). Briefly, microtiter plates (Nalge Nunc International, Rochester, NY, USA) were coated with anti-hIgG3 G3m15 scFv [1.5A10 anti G3m(s,t)] at 5 µg/ml in carbonate buffer, pH 9.6. After washing with phosphate-buffered saline (PBS)/0.05% Tween-20 (PBST), plates were blocked with 1% biotin-free BSA (Carl Roth GmbH, Karlsruhe, Germany) in PBST (1% BSA in PBST). After washing, serum samples and the positive control G3m15 diluted in 1% BSA in PBST were loaded onto the plates and incubated for 2 h. For detection, plates were incubated with biotin-conjugated anti-hIgG3 G3m15 scFv, diluted at 5 µg/ml in 1% BSA in PBST, followed by peroxidase-conjugated streptavidin (Dako Deutschland GmbH, Hamburg, Germany) diluted 1:2,000 in 1% BSA in PBST. For colorimetric detection, plates were developed using TurboTMB (Perbio Science Deutschland GmbH, Bonn, Germany) and stopped with 1 M H_2_SO_4_ solution. OD values were measured at 450 nm using a VICTOR 3™ reader (PerkinElmer Inc., Waltham, MA, USA).

### Determination of the rs4042056 Genotype by KASP Assay

The genotype of the rs4042056 was determined with the KASP method (LGC Genomics GmbH, Berlin, Germany), using a customized assay on a realplex^2^ Mastercycler (Eppendorf AG, Hamburg, Germany), followed by fluorescence end-point reading with an Applied Biosystems 7800 Real-Time PCR system (Life Technologies GmbH, Darmstadt, Germany). For each sample, 10 ng of genomic DNA was used and mixed with KASP reagents according to the manufacturer. The KASP assay contained the allele-specific HEX-conjugated primer 5′-ggc tct tct gcg tga agc-3′, the FAM-conjugated primer 5′-ggc tct tct gcg tga agt-3′, and the common primer 5′-cag gtg gca gca ggg gaa ca-3′.

### Amplification of the rs4042056 Flanking Sequence

The gene sequence flanking the rs4042056 in the human IgG3 gene was amplified by PCR using the forward primer 5′-cag gtg gca gca ggg gaa ca-3′ and the reverse primer 5′-cgg ccg tcg cac tca ttt ac-3′, resulting in a 112 bp product (Figure S1 in Supplementary Material). After agarose gel purification with the QIAquick Gel Extraction Kit (Qiagen, Hilden, Germany), this product was subjected to Sanger sequencing at Eurofins Genomics, Ebersberg, Germany, using the same primers. For PCR, HotStart Phusion polymerase (Life Technologies GmbH, Darmstadt, Germany) was used with Phusion HiFidelity buffer, 1% DMSO and 10 pmol of each primer. PCR program: 98°C for 30 s; 10 cycles of 98°C for 5 s, 75°C (−1.0°C/cycle) for 20 s, 72°C 10 s; 25 cycles of 98°C for 5 s, 65°C for 20 s, 72°C 10 s; final elongation for 5 min at 72°C.

### Statistical Analysis

The open-source software GNU R version 3.15^2^ was used for statistical analysis, together with the package *lme4* for generalized linear mixed effects regression of co-dominant, recessive and dominant genetic effects, including stratification by ethnic cohort. For cohort-wise analyses, Lidstone additive smoothing was applied by addition of a pseudocount of α = 0.5.

For combined analyses of serum and DNA samples, the generalized linear mixed effects regression was extended by using both sample type and ethnic cohort as stratification factors.

For comparison of genotyping results between different methods, Cohen’s weighted kappa was calculated using the function *cohen.kappa* in package *psych*. Hardy–Weinberg equilibrium was checked with the function *HWAlltests* from the R package *HardyWeinberg*.

To compare genotype frequencies determined in sera or DNA samples, we used a logistic regression with the risk allotype/allele as response variable and sample type (or genotyping method), ethnic origin and disease state (PV vs. healthy control) as covariates, including all two- and three-way interactions. For calculation of likelihood-ratio test *p* values, the function *Anova* from R package *car* was applied.

## Results

We collected genomic DNA or serum samples from 516 PV patients and 555 population-matched healthy controls from Germany (*n* = 137 patients, *n* = 259 controls), Iran (*n* = 78 patients, *n* = 90 controls), Turkey (*n* = 148 patients, *n* = 19 controls), and Egypt (*n* = 153 patients, 197 controls) (Table 1). Furthermore, DNA or serum from 173 German patients with BP, another autoimmune blistering skin disease associated with autoantibodies against BP180 (collagen type XVII), was analyzed (Table 2) (20). Separate cohorts were applied for serum and DNA samples.

**Table 1:** IgG3 rs4042056 variation in pemphigus vulgaris by KASP assay.

KASP^a^	Cases				Controls				Recessive^c^		Dominant^c^		Co-dominant^c^	
					
rs4042056	AA	AG	GG	MAF (%)^b^	AA	AG	GG	MAF (%)^b^	OR (95% CI)	*p*	OR (95% CI)	*p*	OR (95% CI)	*p*
Germany	4	0	83	4.6*	0	1	180	0.28	19 (1–350)	0.0074	5.2 (1–27)	0.037	3.2 (1.1–9.5)	0.014
	4.6%	0%	95%		0%	0.55%	99%							

Iran	5	1	72	7.1*	3	0	87	3.3*	1.9 (0.5–7.4)	0.37	2.1 (0.6–7.5)	0.24	1.4 (0.7–2.8)	0.28
	6.4%	1.3%	92%		3.3%	0%	97%							

Turkey	9	1	63	13*	0	0	19	0	5.8 (0.33–100)	0.12	3.4 (0.4–28)	0.19	2.2 (0.6–7.6)	0.15
	12%	1.4%	86%		0%	0%	100%							

Egypt	2	0	99	2*	0	0	126	0	6.4 (0.3–130)	0.16	6.4 (0.3–130)	0.16	2.5 (0.6–12)	0.16
	2%	0%	98%		0%	0%	100%							

Total^g^	20	2	317	6.2	3	1	412	0.84	4.28 (1.6–11.9)	0.0051	3.7 (1.5–9)	0.0038	2.78 (1.0–7.6)	0.047
	5.9%	0.6%	94%		0.7%	0.2%	99%							

**ELISA^d^**	**Cases**				**Controls**						**Dominant^c^**		**ELISA/KASP combined dominant^h^**	
						
**G3m15**	**Positive**	**Negative**		**MAF (%)^e^**	**Positive**	**Negative**		**MAF (%)^e^**			**OR (95% CI)**	***p***	**OR (95% CI)**	***p***

Germany	*1*	*49*		*1.0*	1	77		0.6			1.6 (0.2–15)	0.7	*4.9 (0.9–25.4)*	*0.061*
	*2.0%*	*98.0%*			1.3%	98.7%									
Iran	n.a.^f^	n.a.^f^		n.a.^f^	n.a.^f^	n.a.^f^		n.a.^f^			n.a.	n.a.	n.a.	n.a.

Turkey	4	71		2.7	n.a.^f^	n.a.^f^		n.a.^f^			n.a.	n.a.	n.a.	n.a.
	5.3%	94.7%														

Egypt	2	50		1.9	2	59		1.7			1.6 (0.33–8)	0.54	*2.5 (0.4–13.7)*	*0.298*
	3.8%	96.2%			3.3%	96.7%									

Total^g^	7	170		*2*	3	136		*1.1*			1.75 (0.4–7.6)	0.83	3.6 (1.5–8.8)	0.0041
	4%	96%			2.2%	97.8%									

*^a^The KASP assay directly determined the genotype of rs4042056 SNP that corresponds to the amino acid at residue 435. The minor A allele (g.1053927G>A) allele corresponds to p.Arg435His*.

^b^MAF, minor allele frequency, i.e., frequency of the A allele. An asterisk (*) indicates that the respective genotype distribution is not in Hardy–Weinberg equilibrium.

*^c^Logistic regression (recessive, dominant, and co-dominant models) stratified by ethnic cohort. The 95% confidence interval is given for the odds ratio (OR); the *p* value (*p*) is given for the likelihood ratio test (LR test) on the genotype. For origin-wise models, the raw ORs are shown. In the combined analysis, a generalized linear mixed effects (with implicit adjustment) model with logit link function was applied. In case of 0 values in corresponding data, Lidstone additive smoothing was used to allow calculation of ORs and ensure convergence of the fitting routine*.

*^d^The ELISA detects the G3m15 allotype of IgG3 that is linked to the p.Arg435His variation*.

*^e^MAF, minor allele frequency, i.e., frequency of the allele encoding the G3m15 allotype, inferred assuming Hardy–Weinberg equilibrium*.

*^f^n.a., no serum samples available*.

*^g^Regression calculated with a generalized linear mixed effects model (uncorrelated intercept and genotype effect as random effects)*.

*^h^Logistic regression of combined ELISA and KASP results. The genotyping method was used for stratification in a random effects model (compare table footnote c). For the calculation across all ethnicities, ethnic origin was used together the genotyping method for stratification*.

**Table 2:** IgG3 rs4042056 variation and G3m15 allotype in bullous pemphigoid.

KASP^a^	Cases				Controls				Recessive^c^		Dominant^c^		Co-dominant^c^	
					
rs4042056	AA	AG	GG	MAF (%)^d^	AA	AG	GG	MAF (%)^d^	OR (95% CI)	*p*	OR (95% CI)	*p*	OR (95% CI)	*p*
Germany	1	1	84	0.58*	0	1	180	0.3	6.3 (0.3–160)	0.22	3.2 (0.5–20)	0.2	2.4 (0.7–8.8)	0.16
	1.2%	1.2%	97.8%		0%	0.6%	99.4%							

**ELISA^b^**	**Cases**				**Controls**						**Dominant^c^**		**ELISA/KASP combined dominant^f^**	
						
**G3m15**	**Positive**	**Negative**		**MAF (%)^e^**	**Positive**	**Negative**		**MAF (%)^e^**			**OR (95% CI)**	***p***	**OR (95% CI)**	***p***

Germany	*0*	*87*		*0.0*	1	77		0.6			0.3 (0.01–7.4)	0.42	1.7 (0.2–12.5)	0.61
	0%	100%		1.3%	98.7%								

*^a^Results from the KASP assay, respectively (for comparison of values and footnote comments see Table 1)*.

*^b^Results from the ELISA allotype determination (for comparison of values and footnote comments see Table 1)*.

*^c^Logistic regression (recessive, dominant, and co-dominant models) stratified by ethnic cohort. The 95% confidence interval is given for the odds ratio (OR); the *p* value (*p*) is given for the likelihood ratio test (LR test) on the genotype. In case of 0 values in corresponding data, Lidstone additive smoothing was used to allow calculation of ORs and ensure convergence of the fitting routine*.

^d^MAF, minor allele frequency, i.e., frequency of the A allele. An asterisk (*) indicates that the respective genotype distribution is not in Hardy–Weinberg equilibrium.

*^e^MAF, minor allele frequency, i.e., frequency of the allele encoding the G3m15 allotype, inferred assuming Hardy–Weinberg equilibrium*.

*^f^Logistic regression of combined ELISA and KASP results. The genotyping method was used for stratification in a random effects model (compare table footnote h of Table 1)*.

Designing a PCR-based genotyping method for processing large sample numbers turned out to be challenging. The primer combination had to be specific for the IgG3 subclass and at the same time, should amplify most of the known allotypes and differentiate between the two allelic variants of the rs4042056 SNP (minor allele: g.1053927G>A, Figure 1). For this purpose, the *Kompetitive Allele-Specific PCR* method (KASP™) appeared to be appropriate. The selectivity for the IgG3 gene is provided by a 3′ base (G/A) mismatch of the common primer with the sequence of IgG1, IgG2, and IgG4 gene. This mismatch also suppresses amplification in case of the IGHG3*03 allele [rs79545032, g.105769317A>G, minor allele frequency (MAF) 4% in and 16% in African populations (21)], but 17 of the 19 IgG3 gene alleles listed in the IMGT database were matching the primer sequence sufficiently. To verify the results of the KASP assay we amplified the region flanking the g.1053927G>A variation in the IgG3 gene by PCR in a small number of samples representing the different genotypes. The genotype of this SNP was determined using Sanger sequencing (Figure S1 in Supplementary Material). The comparison showed that both methods were correlating well [Table S1 in Supplementary Material, Cohen’s weighted kappa = 0.62 (95% CI: 0.39–0.85), Fisher’s exact test *p* = 0.0054]. Furthermore, we used a fully saturated logistic regression model to evaluate the dependency of genotype frequencies on disease status, genotyping method, ethnic origin and all possible interactions between those. The likelihood ratio test revealed that only disease status (*p* = 0.002) and ethnic origin (*p* = 0.0095) show a significant influence on the genotype frequency, while the genotyping method—and any interaction of genotyping method with the other covariates—did not have an effect. This confirms that both genotyping methods are equivalent, and it indicates the frequency of the p.Arg435His variation is more common in MENA countries.

For serum samples (*n* = 177 patients, *n* = 136), we used a sandwich ELISA to serologically determine the G3m15 allotype that is described to be linked to the p.Arg435His variation (2) (Table 1). The MAF estimation for the p.Arg435His variation did not differ significantly between the results from the KASP assay (3.3% in cases and 0.5% in controls) and the ELISA (2% in cases and 1.1% in controls). Moreover, the frequencies of the G3m15 allotype and the p.Arg435His variation were different in cases and controls especially in the Egyptian and the Turkish populations. The distribution of genotypes was not in Hardy–Weinberg equilibrium for all cohorts of PV cases and in the Iranian cohort of controls.

To test if our finding for p.Arg435His variation in PV was a general phenomenon for all autoimmune blistering diseases (20) a cohort of *n* = 173 German patients with BP was used. In this cohort of BP patients, no significant association of disease risk with the p.Arg435His or the G3m15 allotype was observed (Table 2). The genotype distribution of BP cases had no Hardy-Weinberg equilibrium.

## Discussion

Allotypic variation has been associated with various autoimmune diseases including autoimmune blistering skin diseases (6, 7). However, the functional role of allotypic variation is mostly unknown with the exception of the p.Arg435His variation which has been demonstrated to affect the half-life of IgG3 (2). We hypothesized that such a functionally relevant variation should be important for the susceptibility of autoantibody-mediated diseases like PV and BP.

High-throughput genotyping of the p.Arg435His variation is hampered by a high variability of the target gene region on the one hand, and a high degree of homology between IgG3 and the other three IgG subclass genes. We were able to design a genotyping method based on KASP technology, but this method did not discriminate heterozygotes from homozygotes. To check the results of this method in a subset of samples representing all detectable genotypes, we used PCR amplification and Sanger sequencing which overall confirmed the validity of the KASP assay. Using the KASP assay, we could demonstrate an association between the p.Arg435His variation and PV in the combined analyses of all ethnic groups, and within the group of German patients. Analysis within the other ethnic groups showed no significant association. This is most probably related to smaller cohort sizes, compared with the German one, because the odds ratio ranged between 1 and 3 in all ethnic cohorts.

As a secondary method for genotyping, we used an ELISA method that detects an amino acid variation which is, according to IMGT databases, linked to the p.Arg435His variation. Using this method alone, we could not find any association with PV disease, which might be explained by the overall lower number of available serum samples in the cohort. However, combining ELISA with KASP genotyping data, using genotyping method and ethnic origin for stratification in a mixed effects logistic regression analysis, reaffirmed the results gained by KASP assay alone. Furthermore, the genotype frequencies of cohorts did not differ significantly between the genotyping methods. Therefore, our results confirm that both genotyping by KASP assay and ELISA lead to equivalent results.

The IgG subclass of autoantibodies in PV is most frequently IgG4, followed by IgG1 (9). IgG2 and IgG3 are found in only sporadically. However, this does not exclude the possibility that the prolonged half-life of IgG3 with the p.Arg435His variation could affect very early preclinical stages of PV. IgG3 is the often first IgG subclass a B cell switches to from IgM, before IgG1 and IgG4 (10). Very early autoantibodies are possibly involved in feedback loops that self-promote autoimmune responses (22). In this scenario, the p.Arg435His variation might enhance these feedback loops by its ability to increase the half-life and biodistribution of these autoantibodies as well as the processing of IgG3-bound autoantigen by enhanced phagocytosis (3). We may speculate that the higher susceptibility to PV associated with the p.Arg435His variation is related to the altered promotion of preclinical PV to the clinically manifest disease. As an alternative explanation, the genomic region flanking the p.Arg435His variation might lead to a changed class switching behavior, as it has been shown for the G3m(b) (Figure 1, G3m5* allotype in current nomenclature) and G3m(g) (Figure 1, G3m21* allotype in current nomenclature) allotype (23). This alternative explanation assumes that the antibody repertoire is changed generally in way that promotes development of PV, but does not require the existence of early IgG3 autoantibodies.

In case of BP, other mechanisms might be important for the disease development. This is not only reflected by differences between PV and BP concerning associations with FcγR polymorphisms (15), but also, as found here, concerning the p.Arg435His variation that does not appears to be associated with BP susceptibility.

A study with individuals from malaria-endemic regions in Benin showed that anti-malaria IgG3 is transferred from pregnant mothers with the p.Arg435His variation to their unborn children and that these children have an improved immunoprotection against malaria (24). In our cohorts, we could find an increased frequency of the minor g.1053927G>A (p.Arg435His) allele in the patient cohorts from Iran and Turkey, in contrast to Egypt and Germany. Iran and Turkey are denoted as malaria-endemic countries from the U.S. Centers of Disease Control, while Egypt and Germany are not (25). A connection between malaria and pemphigus diseases has not been described, yet. Malaria is transmitted by stinging insects. This is remarkable, because in endemic Pemphigus foliaceus in Brazil, a connection to stinging insects has been described: autoantibodies against desmoglein 1 from affected patients cross-react with a salivary protein of a biting fly (26, 27). A genetic test does not allow to distinguish a direct effect of variation on susceptibility to a disease from a population effect. Our data show that the frequency of the p.Arg435His variation is higher in Iran and Turkey, favors the assumption of Dechavanne et al., that this variation could be an evolutionary adaption to malaria (24). Though highly speculative, a link between malaria and PV, be it genetic or environmental, would provide an alternative or additional explanation for the association between PV and the p.Arg435His variation: PV patients might preferably come from malaria-endemic areas within the investigated countries, while controls do not have this preference, leading to an increased frequency of the p.Arg435His variation. Such a population effect cannot be distinguished with the data available. However, a population effect would not imply any direct functional effect of the p.Arg435His variation on PV susceptibility or a theoretical involvement of IgG3 in PV.

The Hardy–Weinberg equilibrium assumption did not hold in any of the PV case cohorts. Next to the above described technical challenges, population effects as described above and the known high degree of consanguinity in the investigated populations could explain this phenomenon (28).

In summary, we found an association of PV but not of BP with a genetic variation that is known to change half-life and effector functions of IgG3. These data suggest that IgG3 may play a role in the pathogenesis of PV or that there may be a link between malaria or malaria-transmitting mosquitoes and PV in MENA countries.

## The German AIBD Genetic Study Group Members

**Alexander Kreuter**, Department of Dermatology, Venereology and Allergology, University of Bochum, Bochum, Germany (current affiliation: Department of Dermatology, Venereology and Allergology, HELIOS St. Elisabeth Klinik Oberhausen, Oberhausen, Germany); **Christos C. Zouboulis**, Department of Dermatology, Venereology, Allergology and Immunology, Dessau Medical Center, Dessau, Germany; **Georg Däschlein**, Department of Dermatology, University of Greifswald, Greifswald, Germany; **Kerstin Steinbrink**, Department of Dermatology, University Medical Center Mainz, University of Mainz, Mainz, Germany; **Manfred Kunz**, Department of Dermatology, Venereology and Allergology, University of Leipzig, Leipzig, Germany; **Nicolas Hunzelmann**, Department of Dermatology, University of Cologne, Cologne, Germany; **Steven Goetze**, Department of Dermatology, University Hospital Jena, Jena, Germany.

## Ethics Statement

This study was carried out in accordance with the recommendations of institutional review board of the University of Lübeck and the review boards of the collaborating centers. The protocol was approved by the institutional review board of the University of Lübeck (File No. 08-156) and the review boards of the collaborating centers. All subjects gave written informed consent in accordance with the Declaration of Helsinki. Samples and demographic data of patients and controls were collected in adherence to ethics and German privacy protection regulations.

## Author Contributions

AR designed research, performed experiments, analyzed data, interpreted results, and wrote the manuscript; SK performed experiments, analyzed data, and wrote the manuscript; SL interpreted results and wrote the manuscript; MF performed experiments and analyzed data; NS, MA, SB, FE-C, ME, RE, RG, CG, EH, BH, WL, WP, RR, MSaeedi, MSárdy, MSticherling, SU, MW, and DZ recruited patients and organized sample collections; SI designed research, interpreted results, and wrote the manuscript; GV designed research, interpreted results, and wrote the manuscript; ES recruited patients, interpreted results, and wrote the manuscript; the German AIBD Genetic Study Group organized sample collections.

## Conflict of Interest Statement

The authors declare that the research was conducted in the absence of any commercial or financial relationships that could be construed as a potential conflict of interest.

## Footnotes

^1^http://www.imgt.org (Accessed: 25 January, 2017).

^2^http://www.R-project.org/ (Accessed: 27 February, 2018).
