# Supplementary material for: The p.Arg435His Variation of IgG3 With High Affinity to FcRn Is Associated With Susceptibility for Pemphigus Vulgaris—Analysis of Four Different Ethnic Cohorts
Source: Front Immunol. 2018 Aug 2;9:1788. doi: 10.3389/fimmu.2018.01788 (PMC6082936; doi:10.3389/fimmu.2018.01788)
Supplement: Supplementary file 1 [file Data_Sheet_1.docx]

## Supplementary Table 1 – Overlap^1^ of KASP assay and PCR/Sanger sequencing results

| **rs4042056 SNP** | | **Genotype by KASP assay (N)** | | |
| --- | --- | --- | --- | --- |
|  |  | **G/G** | **A/G** | **A/A** |
| **Genotype by PCR and Sanger sequencing (N)** | **G/G** | **5** | 1 | 2 |
|  | **A/G** | 3 | **1** | 2 |
|  | **A/A** | 0 | 1 | **10** |

1. The genotype of the rs4042056 was determined either by KASP assay or by Sanger sequencing of a PCR product comprising the region of this SNP. The agreement between KASP and Sanger sequencing is moderate to substantial, as measured by Cohen’s weighted kappa = 0.62 (95% CI: 0.39 -0.85), and significant, as measured by (Fisher’s exact test (p = 0.0054).

# Supplementary Figures

## Supplementary Figure 1 – Sanger sequencing of the rs4042056 SNP

The region containing the rs4042056 SNP (g.1053927G>A, p.Arg435His) was amplified from genomic DNA using PCR with an IGHG3-specific forward primer and a common reverse primer for all IGHG genes (IGHG1, IGHG2, IGHG3 and IGHG4). PCR products were analyzed by Sanger sequencing to identify the genotype of rs4042056. Representative trace plots of dye-terminator Sanger sequencing runs for the three possible genotypes. The SNP position is labeled by a line and a gray shading.
