# Supplementary figures and images for: The p.Arg435His Variation of IgG3 With High Affinity to FcRn Is Associated With Susceptibility for Pemphigus Vulgaris—Analysis of Four Different Ethnic Cohorts
Source: Front Immunol. 2018 Aug 2;9:1788. doi: 10.3389/fimmu.2018.01788 (PMC6082936; doi:10.3389/fimmu.2018.01788)

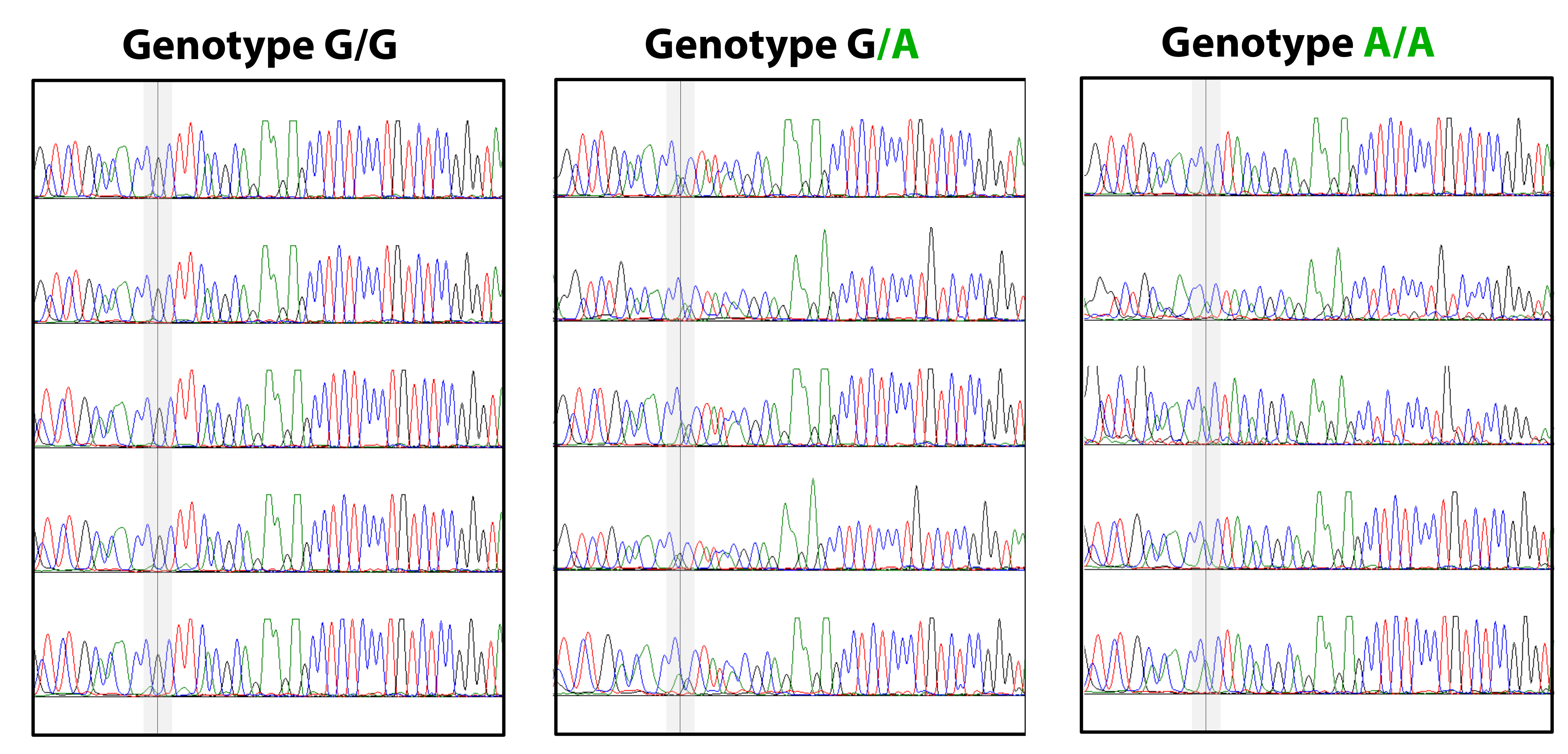

Supplement: Supplementary file 2 [file Image_1.tiff]
